# Pandemic 1918 Influenza Virus Does Not Cause Lethal Infection in Rhesus or Cynomolgus Macaques

**DOI:** 10.1128/jvi.00728-22

**Published:** 2022-08-04

**Authors:** Mable Chan, Meenakshi Tiwary, Helen L. Wu, Nikesh Tailor, Robert Vendramelli, Jonathan Audet, Bryce M. Warner, Kevin Tierney, Alix Albietz, Thang Truong, Kaylie Doan, Alexander Bello, Marnie Willman, Bryan D. Griffin, Patrick W. Hanley, Jamie Lovaglio, David Safronetz, Jim Strong, Jonah B. Sacha, Darwyn Kobasa

**Affiliations:** a Special Pathogens, National Microbiology Laboratory, Public Health Agency of Canada, Winnipeg, Manitoba, Canada; b Vaccine and Gene Therapy Institute, Oregon Health & Science University, Portland, Oregon, USA; c Oregon National Primate Research Center, Oregon Health & Science University, Portland, Oregon, USA; d Vaccine Safety Surveillance, Immunization Branch, Public Health Agency of Canada, Ottawa, Ontario, Canada; e Rocky Mountain Veterinary Branch, Division of Intramural Research, National Institute of Allergy and Infectious Diseases, National Institutes of Health, Hamilton, Montana, USA; f Department of Medical Microbiology and Infectious Diseases, University of Manitoba, Winnipeg, Manitoba, Canada; University of North Carolina at Chapel Hill

**Keywords:** 1918 influenza, cynomolgus macaques, influenza model, rhesus macaque

## Abstract

The 1918 H1N1 influenza pandemic was among the most severe in history, taking the lives of approximately 50 million people worldwide, and novel prophylactic vaccines are urgently needed to prevent another pandemic. Given that macaques are physiologically relevant preclinical models of human immunology that have advanced the clinical treatment of infectious diseases, a lethal pandemic influenza challenge model would provide a stringent platform for testing new influenza vaccine concepts. To this end, we infected rhesus macaques and Mauritian cynomolgus macaques with highly pathogenic 1918 H1N1 influenza virus and assessed pathogenesis and disease severity. Despite infection with a high dose of 1918 influenza delivered via multiple routes, rhesus macaques demonstrated minimal signs of disease, with only intermittent viral shedding. Cynomolgus macaques infected via intrabronchial instillation demonstrated mild symptoms, with disease severity depending on the infection dose. Cynomolgus macaques infected with a high dose of 1918 influenza delivered via multiple routes experienced moderate disease characterized by consistent viral shedding, pulmonary infiltrates, and elevated inflammatory cytokine levels. However, 1918 influenza was uniformly nonlethal in these two species, demonstrating that this isolate is insufficiently pathogenic in rhesus and Mauritian cynomolgus macaques to support testing novel prophylactic influenza approaches where protection from severe disease combined with a lethal outcome is desired as a highly stringent indication of vaccine efficacy.

**IMPORTANCE** The world remains at risk of an influenza pandemic, and the development of new therapeutic and preventative modalities is critically important for minimizing human death and suffering during the next influenza pandemic. Animal models are central to the development of new therapies and vaccine approaches. In particular, nonhuman primates like rhesus and cynomolgus macaques are highly relevant preclinical models given their physiological and immunological similarities to humans. Unfortunately, there remains a scarcity of macaque models of pandemic influenza with which to test novel antiviral modalities. Here, we demonstrate that even at the highest doses tested, 1918 influenza was not lethal in these two macaque species, suggesting that they are not ideal for the development and testing of novel pandemic influenza-specific vaccines and therapies. Therefore, other physiologically relevant nonhuman primate models of pandemic influenza are needed.

## INTRODUCTION

Influenza is one of the major causes of worldwide morbidity and mortality, claiming approximately 500,000 lives annually. Influenza A virus has caused multiple human pandemics over the past century, including in 1918, 1957, 1968, and 2009. The most severe pandemic was the 1918 pandemic caused by an avian-origin H1N1 influenza virus, which was responsible for the deaths of 50 million to 100 million people worldwide. Recently, sporadic bird-to-human transmissions have been reported (1). Furthermore, a few mutations in viral hemagglutinin (HA), which mediates virus binding to host-specific receptors, have manifested efficient aerosol transmission among ferret models (2, 3). This indicates that circulating avian influenza A viruses may have the potential to engender a pandemic.

Our understanding of influenza pathology and transmission is based predominantly on animal model systems. The 1918 influenza strain is highly virulent in small-animal models of mice (4) and ferrets (5) at a dose of 10^6^ TCID_50_ (50% tissue culture infective doses). While small-animal models of influenza possess many advantages, they do not recapitulate human disease pathology, and many human-specific therapeutics cannot be tested. Therefore, the nonhuman primate (NHP) model of influenza is crucial for the development of novel therapeutic and prophylactic treatments for influenza.

The development of an NHP model of influenza that replicates human disease is a critical step for testing candidate treatments. The earliest effort to infect cynomolgus macaques took place in 1997, in a study where macaques were intratracheally infected with very a high dose, 10^7^ TCID_50_, of the influenza virus A/Netherlands/18/94 (H3N2) to test for vaccine effectiveness (6). These H3N2 influenza virus-infected animals displayed no clinical signs of disease; however, the virus did replicate in the respiratory tract to moderate titers. In another report, cynomolgus macaques were challenged by low-dose influenza virus A/Hong Kong/156/97 (H5N1) (highly pathogenic avian H5N1) via multiple routes: intratracheally (2.0 × 10^4^ TCID_50_), tonsillar (2.5 × 10^3^ TCID_50_), and conjunctivas bilaterally (1.25 × 10^3^ TCID_50_) (7, 8). More severe signs of clinical disease and pathology were observed in these H5N1 influenza virus-infected animals; however, mortality from infection was not evaluated as the animals were all euthanized by day 7 postinfection (p.i.) for pathology. Another study used the 1918 influenza virus A/South Carolina/1918 (H1N1) generated by reverse genetics to infect cynomolgus macaques, which led to severe disease pathology, including a high respiration rate, decreased lung function, and increased concentrations of inflammatory cytokines. These findings are based on a non-physiologically relevant high dose (total of 7 × 10^6^ PFU of virus) inoculated through a combination of intratracheal, intranasal (i.n.), intraocular, and oral routes (9). Collectively, these data suggest that the severity of disease depends on the viral strain, inoculum concentration, route of exposure, and host factors. The disease pathology and severity of the 1918 influenza virus strain are currently unknown in cynomolgus macaques at sublethal doses of virus. Therefore, we compared the different doses and routes of inoculation to determine the disease pathology in cynomolgus macaques. Here, we show that even at the highest dose tested, 1918 influenza failed to cause severe disease in both rhesus and Mauritian cynomolgus macaques.

## RESULTS

### 1918 influenza virus causes mild to negligible disease in rhesus macaques.

**FIG 1:**
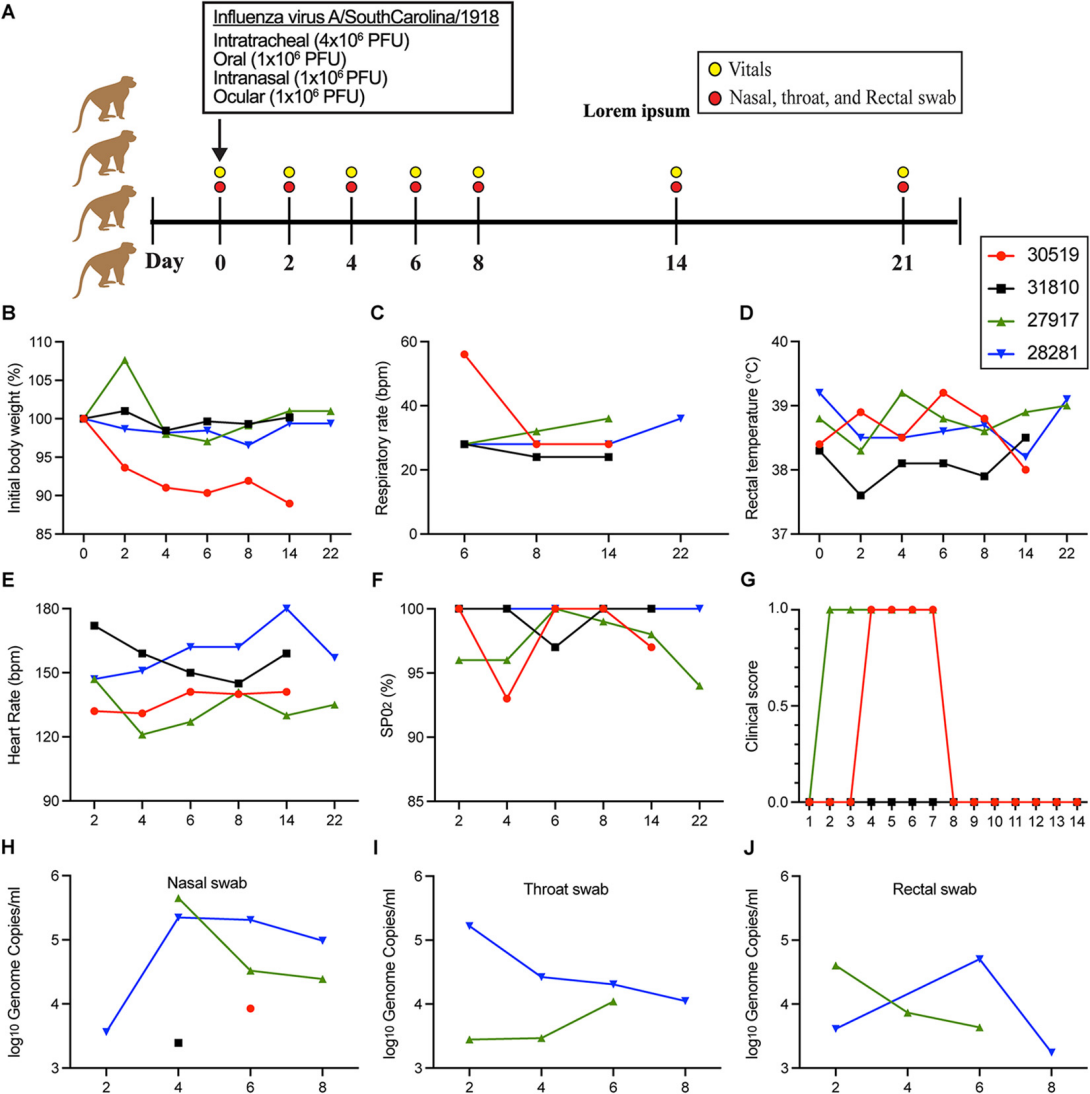
Rhesus macaques infected with 1918 influenza virus. (A) Study overview of four rhesus macaques infected with 7 × 10^6^ PFU of 1918 influenza virus via intranasal, ocular, oral, and intratracheal routes. Blood, vitals, and swabs were collected on the indicated days postinfection. (B to G) Readouts of infection severity for weight loss (B), respiratory rate (C), rectal temperature (D), heart rate (E), oxygen saturation (SPO_2_) (F), and clinical score (G). (H to J) Levels of influenza viral genomes were measured by RT-qPCR in nasal swabs (H), throat swabs (I), and rectal swabs (J). Only samples with values above the limit of detection of the assay (9.3 × 10^2^ genome copies) are shown. bpm, beats per minute.

To determine the susceptibility of rhesus macaques to 1918 influenza, we infected a group of four male rhesus macaques with 7 × 10^6^ PFU of 1918 influenza virus via intranasal, ocular, oral, and intratracheal routes (Fig. 1A). The infectious dose and routes of infection matched those reported previously for this virus in cynomolgus macaques (9). Furthermore, the mouse 50% lethal dose (MLD_50_) of the 1918 influenza virus stock used for macaque infection was determined prior to use to be 10^3.2^ PFU, which was consistent with data from previous reports for this virus (9). We collected blood, bronchoalveolar lavage fluid (BALF), vital signs, and swabs (nasal, throat, and rectal) on days 0, 2, 4, 6, 8, 14, and 22 postinfection (p.i.) and monitored for clinical signs of disease.

Minimal weight loss was observed in all four animals, with a maximum weight loss of about 11% in animal 30519 (Fig. 1B). Only animal 30519 displayed an elevated respiratory rate on day 6 p.i. that resolved by day 8 (Fig. 1C). Animals did not show any signs of fever (Fig. 1D) or significant changes in their heart rate (Fig. 1E). Levels of oxygen saturation (SPO_2_) decreased in animal 30519 to 93% on day 4 p.i. but returned to 100% by day 6 (Fig. 1F). Two animals, 27917 and 30519, showed very mild clinical signs of disease, having a clinical score of 1, while the other two animals remained scoreless for the duration of the study (Fig. 1G). A clinical score of 25 or higher meets the criteria for euthanasia, further underscoring the minimal clinical impact of 1918 influenza infection on rhesus macaques.

Viral loads in the nasal (Fig. 1H), throat (Fig. 1I), and rectal (Fig. 1J) swabs from each of the animals after infection were measured by reverse transcription-quantitative PCR (RT-qPCR). Two animals, 30519 and 31810, had swabs that were positive for viral RNA between days 2 and 8, while the other two animals showed viral RNA in the nasal swab on only one of the sampling days. Of the positive swab samples, infectious virus was found sporadically, ranging from 5 × 10^1^ to 4.13 × 10^3^ PFU/mL (see Table S1 in the supplemental material). Taken together, these results show that 1918 influenza virus replicates variably and poorly in rhesus macaques, with negligible signs of clinical disease.

### Infection of cynomolgus macaques with 1918 influenza virus was nonlethal regardless of the dose and route of exposure.

The limited replication observed in rhesus macaques led us to investigate the replication of 1918 influenza virus in another widely used nonhuman primate species, cynomolgus macaques. Based on previous studies describing a lethal outcome in cynomolgus macaques infected with a dose of 7 × 10^6^ PFU via intranasal, ocular, oral, and intratracheal routes of exposure (9), we hypothesized that lower doses of infection delivered directly to the lung via intrabronchial instillation would uncover the 50% lethal dose (LD_50_).

**FIG 2:**
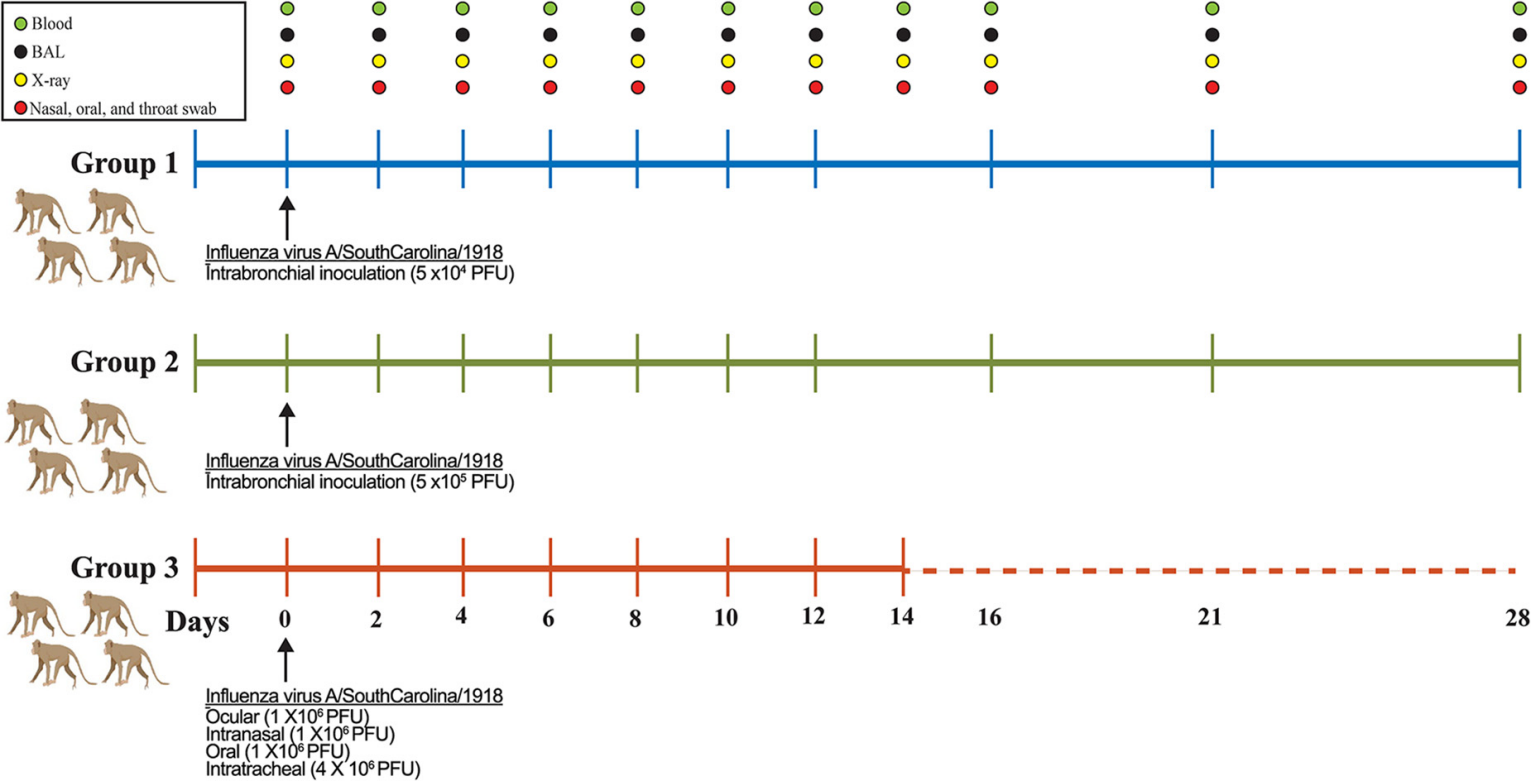
Experimental overview of 1918 influenza virus-infected cynomolgus macaques. Three groups of four cynomolgus macaques were infected with various doses of 1918 influenza virus via multiple routes of inoculation, including intrabronchial, ocular, intranasal, oral, and intratracheal. Sampling days are indicated, and samples collected included blood, bronchoalveolar lavage (BAL) fluid, X ray, and nasal, oral, and throat swabs.

Two groups of cynomolgus macaques were infected with either 5 × 10^4^ PFU (*n* = 4) or 5 × 10^5^ PFU (*n* = 4) of 1918 influenza virus via intrabronchial inoculation. We believed that direct inoculation to the lower respiratory tract would initiate replication sufficient to cause severe disease. Animals were initially sampled every 2 days until day 12 p.i. for blood, bronchoalveolar lavage fluid, radiographs, and swabs to monitor disease progression and viral replication. Other vitals such as weight, heart rate, respiratory rate, oxygen saturation, temperature, and clinical scores were also monitored. A schematic outline of the experimental groups and sampling schedule is shown in Fig. 2. Interestingly, none of the animals in either group developed disease severe enough to warrant euthanasia. As a result of these findings, we included one final group of cynomolgus macaques (*n* = 4) that were infected with a higher dose of 7 × 10^6^ PFU, which matched the dose used previously to elicit a lethal outcome in the same animal model (Fig. 2). Furthermore, we also matched the route of exposure to intranasal, ocular, oral, and intratracheal inoculation. Surprisingly, all four of these animals also survived infection; however, more pronounced disease severity was observed in this group than in the two previous groups.

Hematology data were collected for each of the animals from the three groups (Fig. S1). No differences in the numbers of white blood cells, monocytes, and platelets in the whole blood were noted. The number of lymphocytes increased later on day 10 p.i. for group 1 and group 3, while animals in group 2 appeared to have low levels of lymphocytes detected in the blood. There was also an increase in neutrophils found in the blood of animals from group 2 beginning on day 8 p.i. until day 16 p.i., while group 3 animals showed a spike in neutrophils only on day 8 p.i. Serum biochemistry data were also collected; however, no discernible differences were found across the three groups (Fig. S2).

**FIG 3:**
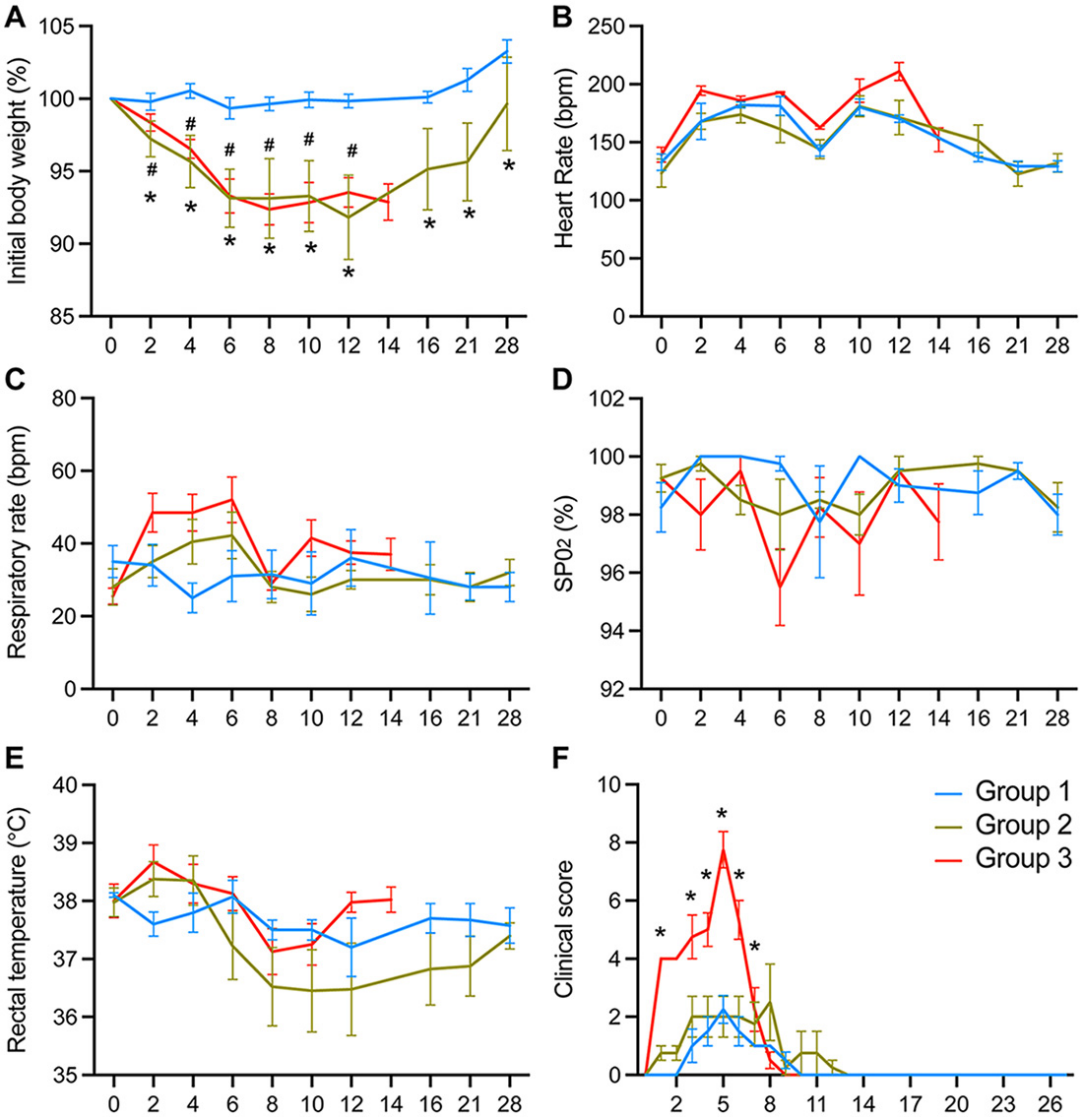
1918 influenza virus infection of Mauritian cynomolgus macaques by multiple doses and routes of exposure. Group 1 animals (*n* = 4) were infected with 5 × 10^4^ PFU via intrabronchial inoculation, group 2 animals (*n* = 4) were infected with 5 × 10^5^ PFU via intrabronchial inoculation, and group 3 animals (*n* = 4) were infected with 7 × 10^6^ PFU via intranasal, ocular, oral, and intratracheal inoculation. Weight loss (A), heart rate (B), respiratory rate (C), oxygen saturation (SPO_2_) (D), rectal temperature (E), and clinical score (F) were monitored for the duration of the study for all animals. Data were compared by two-way ANOVA with Tukey’s multiple-comparison test between the groups. Symbols for group 1 versus group 2 (#) and group 1 versus group 3 (*) represent *P* values of <0.05.

A comparison of the vitals of the three groups of infected cynomolgus macaques showed that the greatest disease severity was observed in the animals from group 3. These animals received the highest dose of inoculum and were infected via multiple routes of exposure, which included exposure to the upper and lower respiratory tracts. Rates of weight loss were comparable between groups 2 and 3, having maximum weight losses of 13.7% and 11.2%, respectively (Fig. 3A). In contrast, animals in group 1 showed a maximum weight loss of 2.9%. Heart rates remained comparable across the three groups (Fig. 3B), while the respiratory rate was noticeably elevated during the acute phase of infection for group 3 (Fig. 3C). Levels of oxygen saturation were the lowest for group 3 on day 6 p.i., with an average of 95.5% (Fig. 3D). Although none of the animals showed any signs of fever (Fig. 3E), those in group 3 had higher clinical scores during the acute phase of infection than did those in groups 1 and 2 (Fig. 3F). Taken together, group 3 animals displayed the greatest disease severity compared to groups 1 and 2. Because group 2 received a 1-log_10_-lower infection dose than group 3, it was not unexpected that the clinical disease observed in group 2 was milder than that in group 3, suggesting that the differences in virus doses and routes of exposure between the two groups contributed to the differences in disease outcomes.

### Viral shedding influenced by the infection dose and route of exposure.

**FIG 4:**
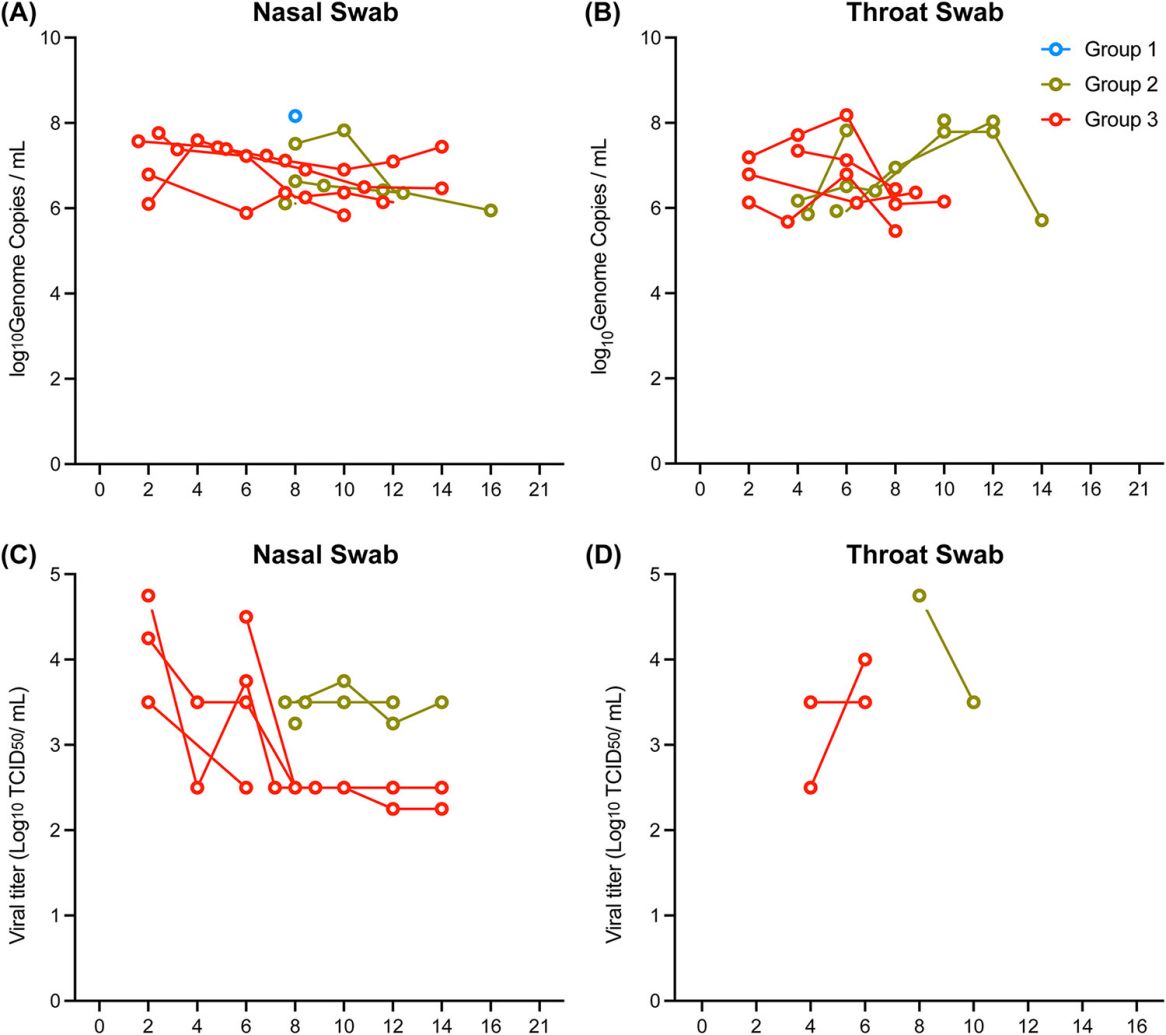
Viral detection in nasal and throat swabs from 1918 influenza virus-infected animals. Three groups of cynomolgus macaques (*n* = 4) were infected with either 5 × 10^4^ PFU (group 1) or 5 × 10^5^ PFU (group 2) of 1918 influenza virus via intrabronchial inoculation or 7 × 10^6^ PFU (group 3) of virus via intranasal, ocular, oral, and intratracheal inoculation. (A and B) Levels of viral RNA were determined by RT-qPCR in the nasal (A) and throat (B) swabs collected from each animal. Only samples with values above the limit of detection of the assay (9.3 × 10^2^ genome copies) are shown. (C and D) Infectious virus in the PCR-positive nasal (C) and throat (D) swabs was measured by TCID_50_ assays.

Levels of viral shedding were monitored in the animals during the course of infection by RT-qPCR. Of the swabs that were collected, nasal and throat swabs were most consistently positive for viral RNA (Fig. 4A and B), while oral swabs were mostly negative for all three groups. Virus replication in group 1 was nearly undetectable, while three out of four animals in group 2 had detectable viral RNA in both nasal and throat swabs. All four animals in group 3 were positive for viral RNA in the nasal and throat swabs, with detection of viral RNA in the nasal swabs as soon as day 2 p.i., compared to the nasal swabs from group 2 animals, which became positive at day 8 p.i.

Infectious viral titers were measured in all RT-qPCR-positive samples by TCID_50_ assays. Infectious virus was present in nasal swabs collected from both group 2 and group 3, although some animals in group 3 reached higher titers than those for group 2 (Fig. 4C). Surprisingly, while many samples were positive for viral RNA in the throat swabs, only a few contained detectable levels of infectious virus for both group 2 and group 3 (Fig. 4D). Similarly, while viral RNA was detected in almost all of the BALF samples collected from all three groups, only one sample from group 2 contained infectious virus (Fig. S3). These results show that a dose of 5 × 10^4^ PFU of 1918 influenza virus was insufficient to induce detectable levels of viral replication in swabs collected from the upper respiratory tract of the animals, and a ≥10-fold-higher dose of virus is likely needed for efficient viral replication. Additionally, exposure to virus in the upper airways of group 3 animals resulted in the earlier detection of infectious virus from the nasal and throat swabs, while initial inoculation into only the lower respiratory tract in group 2 resulted in a delayed spread of the virus to the upper respiratory tract.

Various lung and other respiratory tract tissues, organs, and lymph nodes were collected at the endpoint of each study. Not surprisingly, very limited viral RNA was detected from the tissues that were collected from the group 1 and 2 animals on day 28 p.i., with only 1 and 2 positive tissue samples out of 17, respectively (Fig. S4). For group 3 animals that were necropsied earlier on day 14 p.i., 10 out of 17 tissue samples were positive for viral RNA. All of the tissue samples from every group contained very low levels of viral RNA, having threshold cycle (*C_T_*) values ranging from 34.5 to 37. These results suggest that viral clearance in the infected tissues was occurring as early as day 14 p.i. and was mostly nondetectable by day 28 p.i.

**FIG 5:**
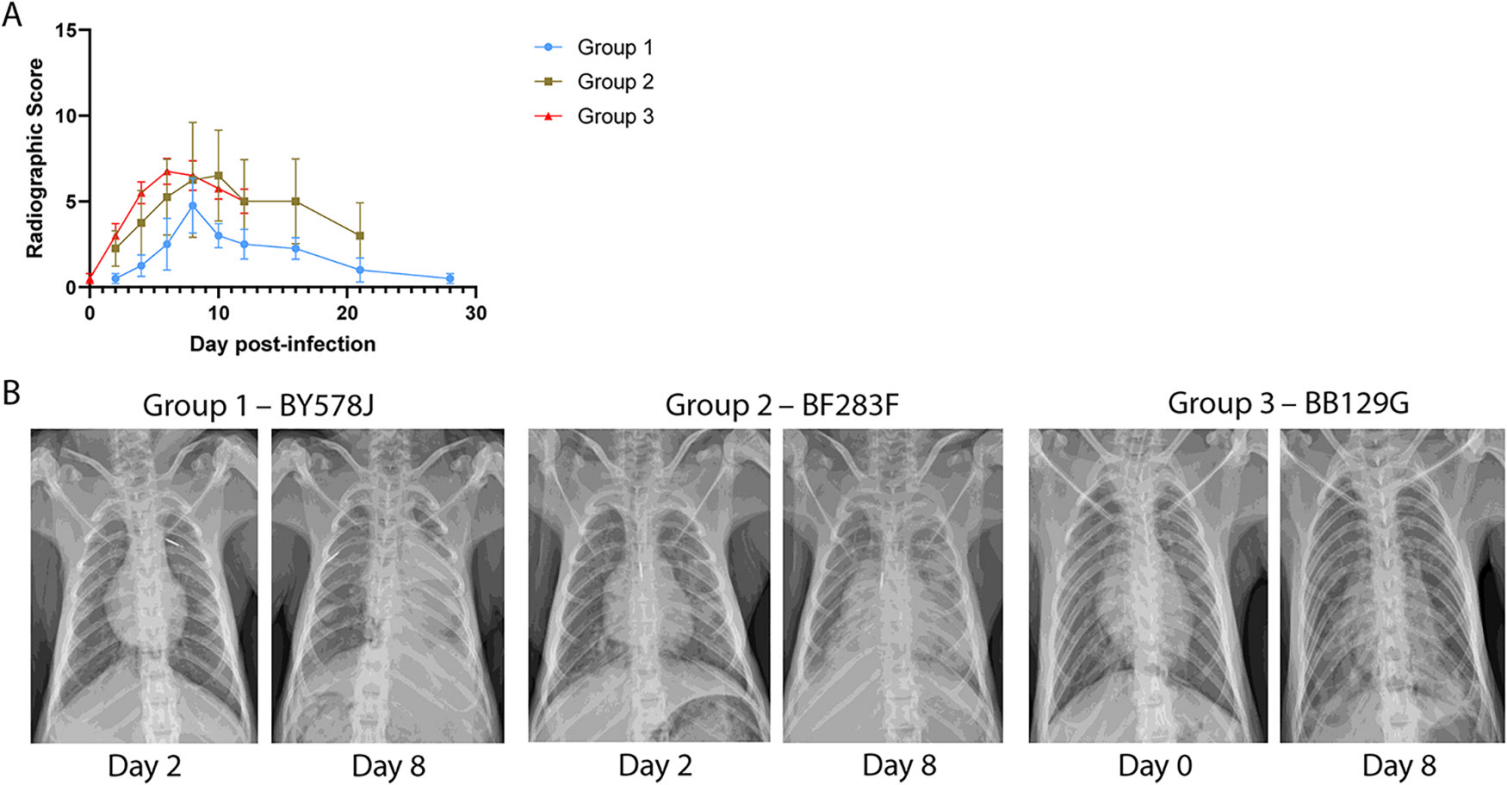
Thoracic radiograph scoring of Mauritian cynomolgus macaques infected with 1918 influenza virus. Three groups of cynomolgus macaques (*n* = 4) were infected with either 5 × 10^4^ PFU (group 1) or 5 × 10^5^ PFU (group 2) of 1918 influenza virus via intrabronchial inoculation or 7 × 10^6^ PFU (group 3) of virus via intranasal, ocular, oral, and intratracheal inoculation. (A) Thoracic radiographs taken on sampling days were scored for the presence of pulmonary infiltrates in each of the lung lobes, and scores were totaled to give a single score on each examination day that could range from 0 to 18. (B) Thoracic radiographs were taken of animals with peak radiographic scores on day 8 postinfection from groups 1, 2, and 3.

Thoracic radiographic evaluation was performed on left lateral and ventrodorsal radiographs collected on examination days during the study. Radiographs were scored for the presence of pulmonary infiltrates in each of the lung lobes, and scores were totaled to give a single score on each examination day that could range from 0 to 18. In the group 1 animals, which had mild clinical signs of disease and minimal viral replication, low to moderate radiograph scores were noted (Fig. 5A). In particular, two animals, AN353J and BY578J displayed moderate scores of 7 and 8, respectively, on day 8 p.i. In comparison to group 1 animals, group 2 animals showed greater weight loss and increased viral detection in the nasal and throat swabs. Two animals showed moderate radiograph scores of 13 and 12 on days 8 and 10 p.i., respectively, while the remaining 2 animals had low scores throughout the study. Surprisingly, only moderate radiograph scores were observed for all animals in group 3, with peak scores of 5 to 8 between days 6 and 8 p.i. Thoracic radiographs from animals in each of the three groups that displayed peak radiographic scores on day 8 p.i. are shown in Fig. 5B. These results show that despite infection using the same dose and route of virus within a group of animals, there were notable differences in the severity of disease observed across the animals. A dose-dependent effect was also observed, where animals in group 1, which received a lower dose of virus, had overall lower radiographic scores than those of the animals in groups 2 and 3, which received 1- to 2-log_10_-larger amounts of virus. Furthermore, it appears that the route of exposure to the virus may influence the degree of radiographic scoring since deeper delivery of virus into the bronchioles of group 2 animals resulted in higher peak scores than those for animals from group 3, which received delivery to the upper respiratory tract and trachea.

**FIG 6:**
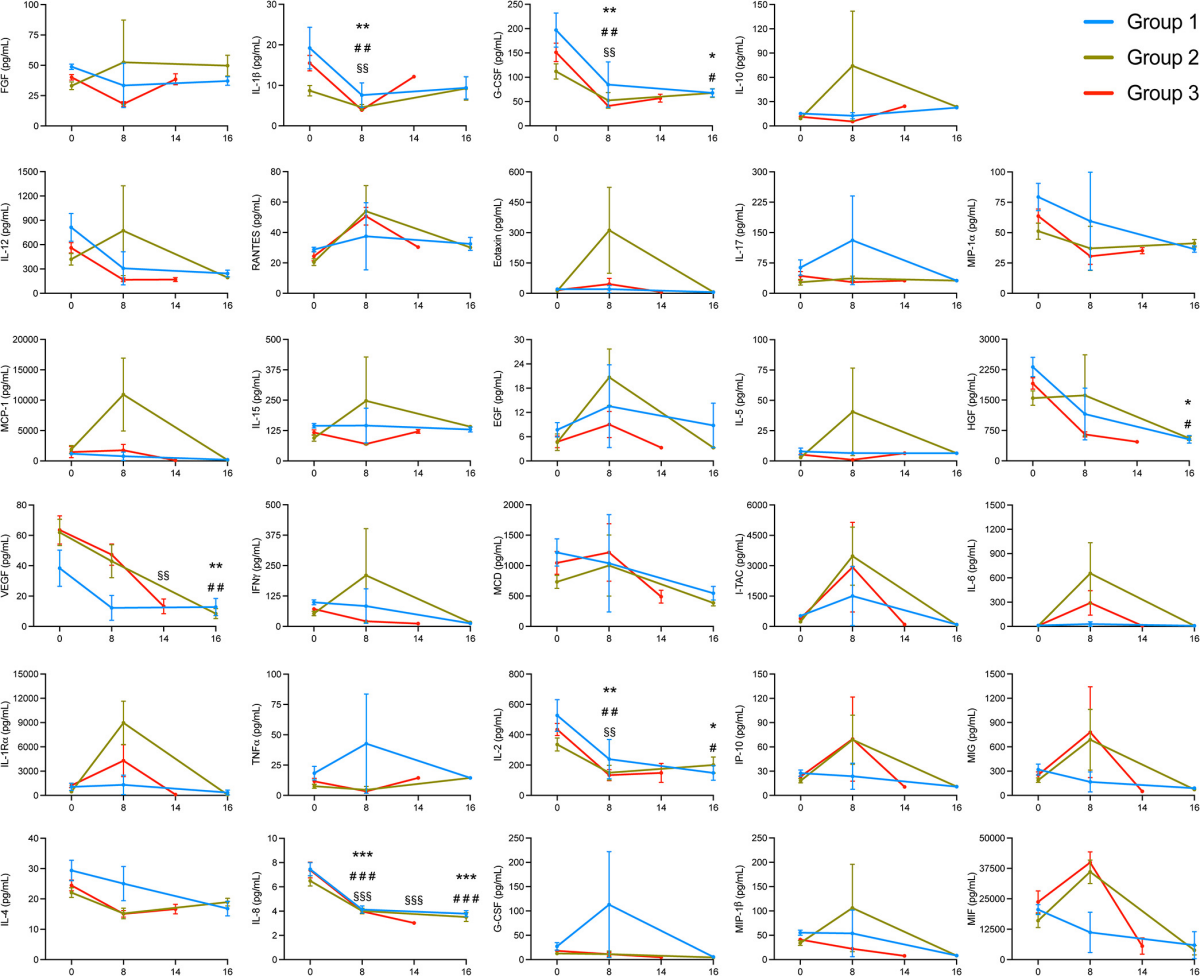
Cytokine expression in the bronchoalveolar lavage fluid samples of Mauritian cynomolgus macaques infected with 1918 influenza virus. Three groups of cynomolgus macaques (*n* = 4) were infected with either 5 × 10^4^ PFU (group 1) or 5 × 10^5^ PFU (group 2) of 1918 influenza virus via intrabronchial inoculation or 7 × 10^6^ PFU (group 3) of virus via intranasal, ocular, oral, and intratracheal inoculation. Individual cytokine expression levels were determined using a cytokine monkey magnetic 29-plex panel. Data were analyzed by two-way ANOVA with Tukey’s multiple-comparison test. Data are shown as means and SEM. *, #, and § represent significance within group 1, group 2, and group 3, respectively, when each time point was compared with day 0. One, two, and three symbols represent *P* values of <0.05, <0.01, and <0.001, respectively.

The expression levels of 29 different cytokines and chemokines in the BALF were measured in all of the animals to further understand whether inflammatory or immune-stimulating cytokines differed across the groups. The majority of the analytes measured displayed similar trends across all three groups, including fibroblast growth factor (FGF), interleukin-1β (IL-1β), IL-12, RANTES, macrophage inflammatory protein 1α (MIP-1α), IL-15, epidermal growth factor (EGF), IL-5, hepatocyte growth factor (HGF), vascular endothelial growth factor (VEGF), interferon gamma (IFN-γ), macrophage-derived chemokines (MDC), interferon-inducible t cell alpha chemoattractant (I-TAC), IL-2, IL-4, IL-8, and MIP-1β (Fig. 6). However, there were a few that were noticeably elevated at day 8 p.i. in the animals from group 2 and group 3, which include IL-6, IL-1 receptor antagonist (IL-1RA), interferon gamma-induced protein 10 (IP-10), monokine induced by gamma interferon (MIG), and macrophage migration inhibitory factor (MIF). In particular, the expression levels of IL-6 and IL-1RA were higher in group 2 than in group 3 at 8 days p.i. Furthermore, levels of eotaxin and monocyte chemoattractant protein 1 (MCP-1) were elevated only in group 2 on day 8 p.i. Of these cytokines and chemokines, MCP-1, IP-10, MIG, and MIF are proinflammatory. These results suggest that more inflammation was occurring in the group 2 and group 3 animals than in group 1.

Interestingly, unique to group 1 was the elevated expression of tumor necrosis factor alpha (TNF-α), granulocyte colony-stimulating factor (G-CSF), and IL-17 at day 8 p.i. TNF-α and IL-17 are proinflammatory cytokines, while G-CSF is a chemoattractant for neutrophils. In contrast, nearly baseline levels of TNF-α, G-CSF, and IL-17 were observed for group 2 and group 3 animals on day 8 p.i. These results suggest that the activation of a different set of proinflammatory cytokines occurred in animals from group 1 compared to groups 2 and 3 and that these differences may have contributed to the varying disease severity observed among the groups.

## DISCUSSION

This study compared the disease progression of 1918 influenza virus in two different nonhuman primate models of disease. Our initial assessment of rhesus macaques showed that 1918 influenza virus infection results in insignificant to mild clinical disease despite inoculation with a high titer and exposure via multiple routes. Inconsistent viral replication was observed in these animals, with only two out of four animals showing detectable levels of viral RNA during the acute phase of infection. Taken together, these results show that 1918 influenza virus replicates variably and poorly in rhesus macaques, resulting in minimal to negligible signs of clinical disease. These results may be due to the lower expression levels of sialyl-α-2,6-Gal saccharides, the main receptor for human influenza A viruses, in the lungs of rhesus macaques (10).

We next investigated whether cynomolgus macaques are a lethal model for 1918 influenza virus infection. Previous publications have described cynomolgus macaques to show severe clinical signs of disease warranting euthanasia of the animals between days 8 and 9 p.i. (9, 11). Based on these studies, we aimed to determine what the 50% lethal dose was, starting with an inoculation titer that was 2 log_10_ PFU lower than the one reported previously (9). Surprisingly, despite testing inoculation doses from 5 × 10^4^ up to 7 × 10^6^ PFU, where the latter matched the dose and route reported previously (9), we did not see signs of severe clinical disease that warranted euthanasia.

In general, we observed a milder outcome in our infected cynomolgus macaques, even though we used the same infection dose and routes of exposure as the ones reported previously (9). Although high levels of virus replication, the presence of pulmonary infiltrates, and increases in respiratory rates were observed in our study, the clinical scores of the animals did not meet our criteria for euthanasia. Similar to previously reported findings (9), we saw elevated levels of IL-6, MCP-1, and RANTES in our infected animals. Additionally, we saw increased expression levels of other proinflammatory cytokines and chemokines, such as IP-10, MIG, and MIF, in our group 2 and group 3 infected animals on day 8 p.i. Increased expression of IL-6 has been described by others to be correlated with clinical disease symptoms caused by influenza virus infection (12–15), and MCP-1 has been described to be released in response to influenza virus infection (12, 16, 17). While our infected cynomolgus macaques followed a disease progression similar to those reported previously, the diminished disease severity in our animals did not warrant euthanasia. These results suggest that other underlying differences in our studies contributed to the variation in outcomes.

To date, there are very limited studies on the pathogenicity of 1918 influenza virus in nonhuman primates (9, 11). In those two studies, there were five cynomolgus macaques infected with 1918 influenza virus and otherwise not treated. In an initial study of viral pathogenesis, three animals were euthanized on day 8 p.i. due to the severity of clinical illness requiring humane euthanasia (9). In the second study, to evaluate the antiviral oseltamivir, the two untreated infection controls were euthanized on days 8 and 9 p.i. based on clinical scores reaching the criteria for euthanasia (11). The animals in those studies came from a Canadian colony that was depopulating the older macaques, and the ages of the animals used in those studies ranged from 9 to 19 years. Out of the 12 animals used in our study, only 4 animals were between the ages of 9 and 12 years, while the rest were 3 years old and younger. Immune senescence in aged macaques has been described previously and suggests that the deterioration of both innate and adaptive immunity as animals age may influence how well they fight off infections (18, 19). In cynomolgus macaques infected with H7N9 influenza virus, more severe symptoms were observed in aged animals than in young animals, with higher expression levels of cytokine and chemokines in the lungs of young animals (20). It is possible that the younger animals used in our study had better-equipped immune systems that were able to handle 1918 influenza virus infection, resulting in a nonlethal outcome. Nevertheless, Mauritian cynomolgus macaques have been reported to succumb uniformly to aerosolized H5N1 influenza, suggesting that these animals may hold promise for aerosolized influenza challenge (20, 21 Simon Barratt-Boyes, personal communication).

Another important difference of our studies is the genetic diversity of the animals. While the origins of the animals in the previous studies are unknown, the majority of cynomolgus macaques used for biomedical research in the United States come from breeding farms in China that capture wild founders from Indonesia, the Philippines, Cambodia, Laos, and Vietnam (22). Furthermore, evidence of interbreeding between cynomolgus macaques and rhesus macaques in Indochina has been described (23, 24). The cynomolgus macaques used in our study are of Mauritian origin. In a genetic screen of several populations of cynomolgus macaques frequently used for biomedical research, Mauritian macaques had the lowest allelic diversity (22). These genetic differences among species may also account for the variation in disease severity observed due to 1918 influenza virus infection.

In summary, while cynomolgus macaques may not be a uniformly lethal model for 1918 influenza virus infection, these animals are susceptible to a high-dose infection that will induce clinical signs of disease that are similar to those of human infection. In contrast, rhesus macaques should not be used to study 1918 influenza virus infection, as infected animals displayed negligible signs of clinical disease. Factors such as the age, origin, and genetic diversity of the cynomolgus macaques may influence the overall severity of infection and should be taken into consideration if these animals are to be used as a model for therapeutic or vaccine testing.

## MATERIALS AND METHODS

### Virus and cells.

Influenza virus A/South Carolina/1918 (H1N1) was generated by reverse genetics (9) and handled in biosafety level 4 (BSL-4) containment at the National Microbiology Laboratory (NML). Sequences of the 1918 influenza viral segments were based on data reported under GenBank accession numbers DQ208309, DQ208310, DQ208311, AF117241, AY744935, AF250356, AY130766, and AF333238. 1918 influenza virus was cultured using Madin-Darby canine kidney (MDCK; ATCC, Manassas, VA, USA) cells. MDCK cells were grown in minimum essential medium (MEM; HyClone) supplemented with 5% fetal bovine serum (FBS; HyClone) and 1× l-glutamine (l-Glu; Gibco, Life Technologies, Grand Island, NY, USA).

A passage 2 (P2) virus stock was prepared using MEM supplemented with 0.1% bovine serum albumin (BSA) (fraction V; HyClone), 1× l-glutamine, and 1 μg/mL *N*-tosyl-l-phenylalanine chloromethyl ketone (TPCK)-treated trypsin (Sigma-Aldrich). This stock was used for animal inoculation. The mouse 50% lethal dose (MLD_50_) for this stock was determined previously to be 10^3.2^ PFU (9); this value was confirmed prior to the use of the stock for macaque infection.

### Mouse 50% lethal dose.

To determine the MLD_50_ of the 1918 influenza virus, 6-week-old female BALB/c mice (Charles River, Canada) were anesthetized using isoflurane and infected intranasally (i.n.) with 10-fold serial dilutions of virus (*n* = 5 mice/dilution) in 50 μL MEM supplemented with 0.1% BSA. Mice were monitored daily and scored for signs of illness, including weight loss, ruffled fur, hunched posture, and rapid or labored breathing. Mice were euthanized when they lost ≥25% of their initial weight. The MLD_50_ value was calculated using the method of Reed and Muench (25).

### Nonhuman primates.

In the first NHP study, four male rhesus macaques (aged 4 to 7 years) were transferred to the NML in Canada from the Oregon National Primate Research Center in the United States. Macaques were free of *Cercopithecine herpesvirus 1*, D-type simian retrovirus, simian T-lymphotropic virus type 1, and Mycobacterium tuberculosis and confirmed to be serologically negative for H1N1 influenza via hemagglutination inhibition (HI) assays. Animals were screened for antibodies against 2009 H1N1 pandemic influenza viruses, which are antigenically related to the 1918 pandemic virus, by standard HI assays using turkey red blood cells as previously described (26). Animals were allowed to acclimatize at the NML Veterinary Technical Services under BSL-2 containment for at least 7 days and were then transferred into BSL-4 containment for infection. Animals were infected with a P2 stock of 1918 influenza virus diluted in MEM at a dose of 7 × 10^6^ PFU via multiple routes: 1 mL ocular (0.5 mL/eye), 1 mL intranasal (0.5 mL/naris), 1 mL oral, and 4 mL intratracheal. After infection, animals were scored daily, and weight loss, temperature, respiratory rate, heart rate, and levels of SPO_2_ were monitored. Animals were sampled once before infection and then on days 2, 4, 6, 8, 14, and 22 postinfection. Swabs (nasal, throat, and rectal) and serum samples were collected to monitor viral loads. Thoracic radiographs, serum biochemistry, and hematology were performed.

In the second NHP study, 12 Mauritian cynomolgus macaques confirmed to be serologically negative for H1N1 influenza via standard HI assays as described above were purchased from an accredited vendor, randomly divided into three groups (*n* = 4), and subsequently infected with the same 1918 influenza virus stock as the one used in rhesus macaques. Each group contained two males and two females, and their ages varied from 5 months to 12.5 years. Group 1 was infected with a dose of 5 × 10^4^ PFU by intrabronchial inoculation to the left side. Group 2 was infected with a dose of 5 × 10^6^ PFU by intrabronchial inoculation to the left side. Group 3 was infected with a dose of 7 × 10^6^ PFU via multiple routes: 1 mL ocular (0.5 mL/eye), 1 mL intranasal (0.5 mL/naris), 1 mL oral, and 4 mL intratracheal. After infection, animals were scored daily, and weight loss, temperature, respiratory rate, heart rate, and levels of SPO_2_ were monitored for the duration of each study. Animals from groups 1 and 2 were euthanized on day 28, while animals from group 3 were euthanized on day 14. Blood, serum, and swab samples were collected on days 0, 2, 4, 6, 8, 10, 14, 16, 21, and 28 for analyses of viral loads, serum biochemistries, hematology, and cytokine responses. Bronchoalveolar lavage fluid (BALF) was collected on days 0, 8, 14, 16, 21, and 28. Thoracic radiographs (left lateral and ventrodorsal) were taken on sampling days to monitor for pulmonary infiltrates. Tissues from the lungs, bronchiole lymph nodes, tonsils, bronchi, trachea, bronchioles, liver, kidney, spleen, and heart were also collected on necropsy days for analysis of viral loads.

### Bronchoalveolar lavage.

Anesthetized animals were placed in the prone position and intubated. Following intubation, animals were placed in left lateral recumbency, and a sterile tracheal suction catheter was inserted into the endotracheal tube through the trachea and into the bronchi. Sterile phosphate-buffered saline (PBS) (8 to 11 mL) was infused into the catheter, followed by aspiration and collection of the BALF. The same procedure was repeated two to three times. A pulse oximeter was used throughout the procedure to monitor the animal’s heart rate and oxygen saturation, and the animal was provided supplemental oxygen. BALF was spun down at 600 × *g* for 10 min to separate cells and BALF for 1918 influenza virus detection.

### Clinical and thoracic radiograph scoring.

Clinical scoring was assessed based on changes in the cardiovascular, respiratory, digestive, nervous, and musculoskeletal systems of the animals from their normal preinfection baseline values (27). Scoring was evaluated by animal technicians based on parameters such as posture, temperature, weight, respiration, feces, urine, food, water, recumbency, attitude, and central nervous system (CNS) signs. Clinical scores for each animal may range from 0 to 64 on each examination day, and euthanasia occurs when a total score of 25 or higher is reached on any examination day.

Radiographs are evaluated and scored for the presence of pulmonary infiltrates by two board-certified clinical veterinarians according to a standard scoring system (28). Briefly, each lung lobe (upper left, middle left, lower left, upper right, middle right, and lower right) is scored individually based on the following criteria: 0 for a normal examination; 1 for mild interstitial pulmonary infiltrates; 2 for moderate interstitial pulmonary infiltrates, perhaps with partial cardiac border effacement and small areas of pulmonary consolidation (alveolar patterns and air bronchograms); and 3 for pulmonary consolidation as the primary lung pathology, seen as a progression from grade 2 lung pathology.

Thoracic radiograph findings are reported as a single radiograph score for each animal on each examination day. To obtain this score, the scores assigned to each of the six lung lobes are added and recorded as the radiograph score for each animal on each examination day. Scores may range from 0 to 18 for each animal on each examination day.

### Blood biochemistry and hematology.

Whole-blood samples were analyzed for complete blood counts using the HM5 hematology system (Abaxis Veterinary Diagnostics). Levels of white blood cells, lymphocytes, monocytes, neutrophils, and platelets were determined. A VetScan VS2 chemistry analyzer (Abaxis Veterinary Diagnostics) and VetScan comprehensive diagnostic profile reagent rotors (Abaxis, Union City, CA, USA) were used to measure the levels of alkaline phosphatase (ALP), alanine transaminase (ALT), amylase (AMY), blood urea nitrogen (BUN), albumin (ALB), creatinine (CRE), and total bilirubin (TBIL).

### Viral assays.

Viral loads in blood, swab, and tissue samples were determined by RT-qPCR detecting the 1918 M gene as described previously (29). A QIAamp viral RNA minikit (Qiagen) was used to extract viral RNA from blood and swab samples according to the manufacturer’s protocol. Tissue samples were stored in RNAlater (Thermo Fisher Scientific), and RNA was extracted using an RNeasy Plus minikit (Qiagen). TaqPath 1-step multiplex master mix (Applied Biosystems) was used for RT-qPCR, reaction mix setup, and cycling conditions as recommended by the manufacturer, and detection was performed with a QuantStudio 5 instrument (Applied Biosystems).

TCID_50_ assays were performed on all RT-qPCR-positive samples using MDCK cells. Tissue samples were weighed and homogenized in 1 mL of MEM–0.1% BSA–l-Glu–2× penicillin-streptomycin (PS) with a 5-mm stainless steel bead using a Bead Ruptor Elite tissue homogenizer (Omni) for 30 s at a frequency of 4 m/s. Cell debris was pelleted by centrifugation at 1,500 × *g* for 10 min, and the supernatant was used for TCID_50_ assays. Samples were serially diluted (1:10) in MEM supplemented with 0.1% BSA, l-Glu, and TPCK-trypsin (MEM–BSA–l-Glu–trypsin), and 100 μL of each dilution was added to a 96-well plate of MDCK cells in triplicate. The plates were incubated at 37°C with 5% CO_2_, and the presence of cytopathic effect (CPE) was observed at 4 to 5 days postinfection. The TCID_50_ titer per milliliter or gram of tissue was determined using the method of Reed and Muench (25).

### Cytokine assays.

Cytokine responses in BALF samples from NHPs were examined using the cytokine monkey magnetic 29-plex panel for the Luminex platform (Thermo Fisher Scientific). Cytokines that were analyzed included G-CSF, IFN-γ, IL-10, IL-12, IL-17A, IL-2, IL-4, IL-6, IL-8, MCP-1, MIP-1α, MIP-1β, RANTES, TNF-α, EGF, eotaxin, FGF-2, granulocyte-macrophage colony-stimulating factor (GM-CSF), HGF, IL-1β, IL-1RA, IL-15, IL-5, IP-10, I-TAC, MDC, MIF, MIG, and VEGF-A. The procedure was performed according to the manufacturer’s recommendations; BALF samples were tested undiluted, and test plates were run using a Luminex Magpix instrument.

### Statistics.

Data are presented as the means and standard errors of the means (SEM) for each group. Statistical analysis was performed within the group and between the groups at each time by two-way analysis of variance (ANOVA) with Tukey’s multiple-comparison tests using GraphPad Prism software v9.2 (GraphPad, La Jolla, CA). Significance at a *P* value of <0.05 is marked by *, #, and § in the figures.
