# Supplementary material for: Pandemic 1918 Influenza Virus Does Not Cause Lethal Infection in Rhesus or Cynomolgus Macaques
Source: J Virol. 2022 Aug 4;96(16):e00728-22. doi: 10.1128/jvi.00728-22 (PMC9400491; doi:10.1128/jvi.00728-22)
Supplement: Supplemental file 1 — Fig. S1 to S4; Table 1. Download jvi.00728-22-s0001.pdf, PDF file, 0.5 MB [file jvi.00728-22-s0001.pdf]

Supplemental Figure S1

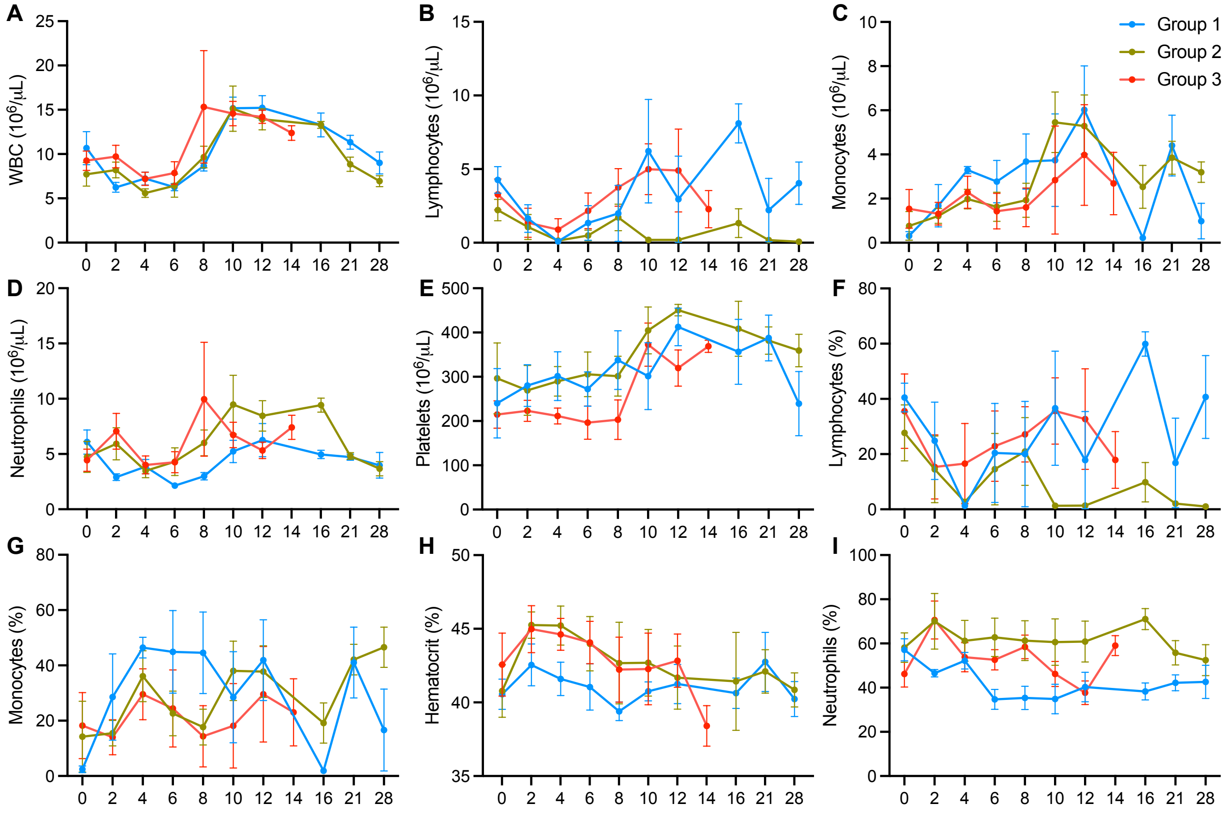

Supplemental Figure S1. Hematology data from 1918 influenza virus infected Mauritian cynomolgus macaques. Group 1 animals (n = 4) were infected with 5 x 10^4 PFU via intrabronchial inoculation, Group 2 (n = 4) were infected with 5 x 10^5 PFU via intrabronchial inoculation, and Group 3 (n = 4) were infected with 5 x 10^7 PFU via intranasal, ocular, oral, and intratracheal inoculation. Complete blood counts of white blood cells (WBC), lymphocytes, monocytes, neutrophils, and platelets are shown.

Supplemental Figure S2

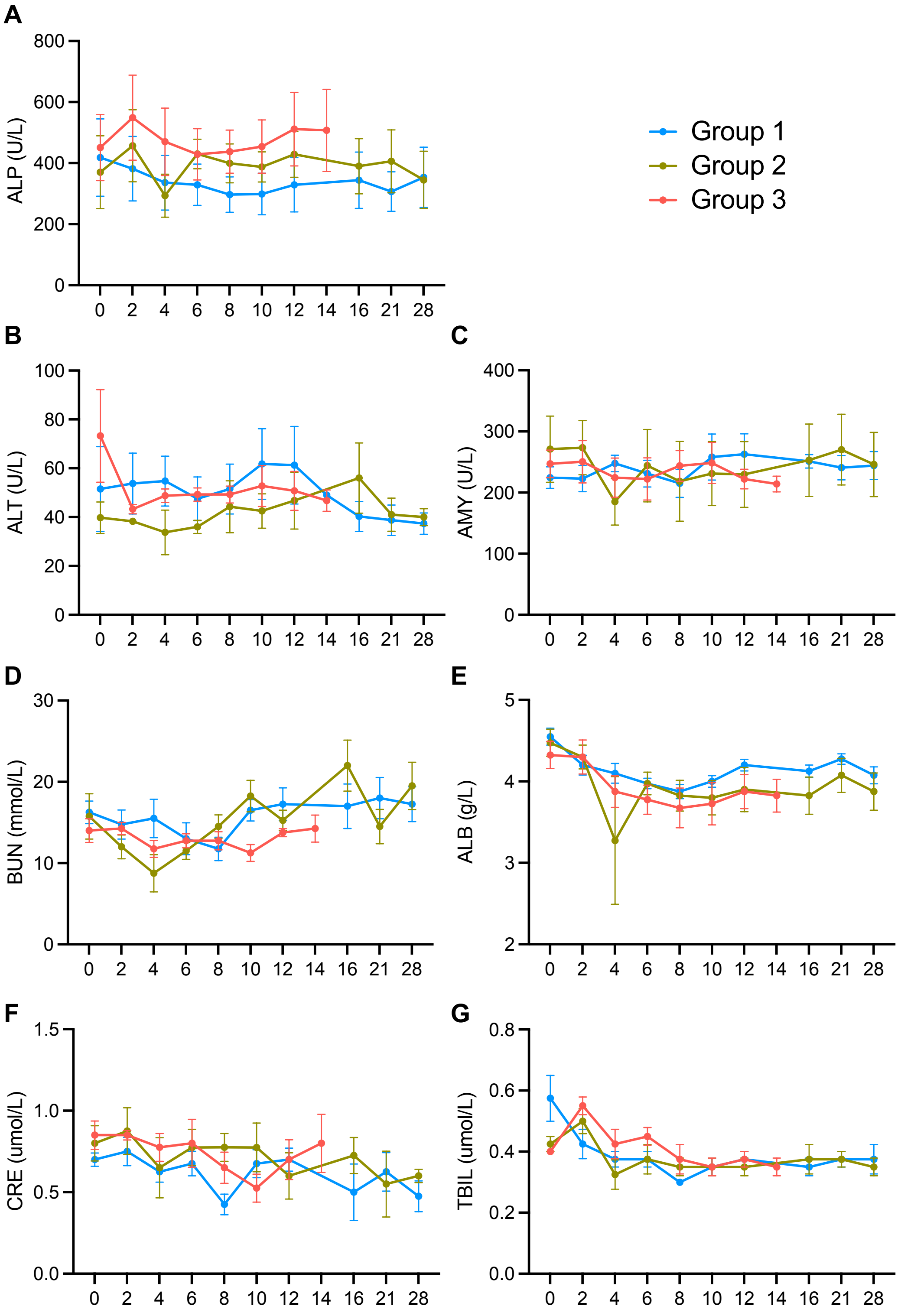

,

Supplemental Figure S2. Blood biochemistry data from 1918 influenza virus infected

Mauritian cynomolgus macaques. Group 1 animals (n = 4) were infected with 5 x 10^4 PFU via intrabronchial inoculation, Group 2 (n = 4) were infected with 5 x 10^5 PFU via intrabronchial inoculation, and Group 3 (n = 4) were infected with 5 x 10^7 PFU via intranasal, ocular, oral, and intratracheal inoculation. A VetScan VS2 chemistry analyzer (Abaxis Veterinary Diagnostics) along with VetScan Comprehensive Diagnostic Profile reagent rotors (Abaxis, Union City, CA, USA) were used to measure levels of ALP, ALT, AMY, BUN, ALB, CRE, TBIL.

Supplemental Figure S3

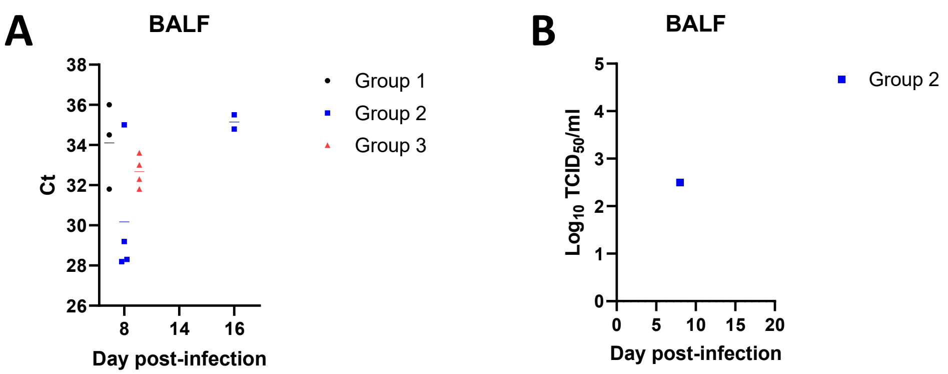

Supplemental Figure S3. Viral replication in the bronchioalveolar lavage fluid (BALF). Three groups of Mauritian cynomolgus macaques (n = 4) were infected with either 5 x 10^4 PFU (Group 1) or 5 x 10^5 PFU (Group 2) of 1918 influenza virus via intrabronchial inoculation, or 7 x 10^6 PFU (Group 3) of virus via intranasal, ocular, oral, and intratracheal inoculation. (**A**) Viral RNA in the BALF was detected by RT-qPCR, Ct values are shown. (**B**) Levels of infectious virus was measured by $TCID_{50}$/ml assays.

Supplemental Figure S4

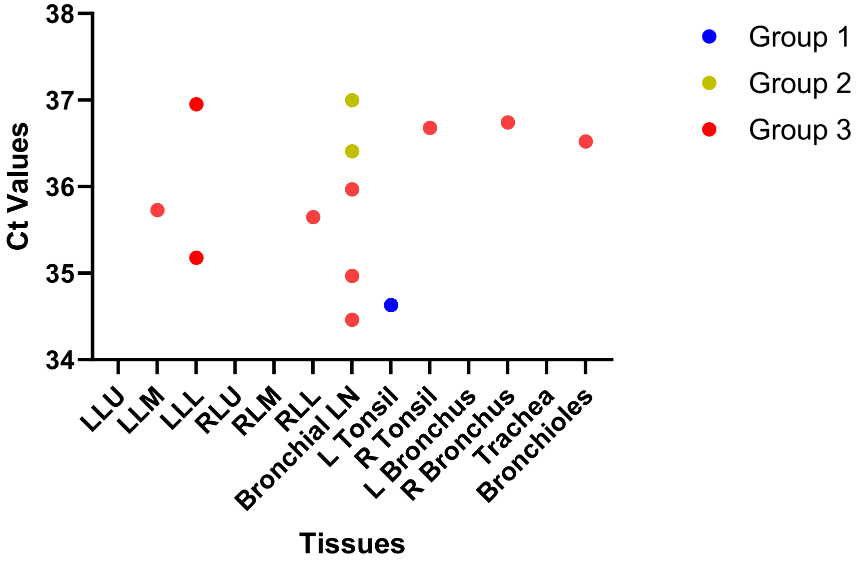

Supplemental Figure S4. Viral RNA detection in tissues collected from 1918 influenza virus infected Mauritian cynomolgus macaques by RT-qPCR. Three groups of cynomolgus macaques (n = 4) were infected with either 5 x 10^4 PFU (Group 1) or 5 x 10^5 PFU (Group 2) of 1918 influenza virus via intrabronchial inoculation, or 7 x 10^6 PFU (Group 3) of virus via intranasal, ocular, oral, and intratracheal inoculation. Group 1 and 2 animals were necropsied on day 28 pi and Group 3 animals were necropsied on day 14 pi. Tissues collected included: Left lung upper lobe (LLU), left lung middle lobe (LLM), left lung lower lobe (LLL), right lung upper lobe (RLU), right lung middle lobe (RLM), right lung lower lobe (RLL), bronchial lymph nodes, left and right tonsil, left and right bronchus, trachea, and bronchioles.

Supplemental Table 1

Animal ID	Swab	Day 2	Day 4	Day 6	Day 8
27917	Nasal	-	4.15×10^3	ND	1.50×10^2
	Throat	ND	ND	ND	-
	Rectal	ND	ND	ND	-
28281	Nasal	1.80×10^3	ND	ND	5.00×10^1
	Throat	ND	ND	ND	ND
	Rectal	ND	-	ND	ND
30519	Nasal	-	-	7.50×10^1	-
	Throat	-	-	-	-
	Rectal	-	-	-	-
31810	Nasal	-	2.48×10^3	-	-
	Throat	-	-	-	-
	Rectal	-	ND	-	-

– : Swab was negative by RT-qPCR, no plaque assay performed
ND: No plaques detected. Limit of detection of the assay was 1×10^3 PFU/mL.

Supplemental Table 1. Infectious virus titers in swabs collected from rhesus macaques infected with 1918 influenza virus. A group of 4 animals were infected with 7 x 106 PFU of 1918 influenza virus via intranasal, ocular, oral, and intratracheal routes. Nasal, throat, and rectal swabs were collected on days 0, 2, 4, 6, 8, 14, and 22 post-infection. Only swabs that were RT-qPCR positive for viral RNA were selected for further plaque assay to measure levels of infectious virus (PFU/mL). "-" swab was negative by RT-qPCR, no plaque assay was performed for sample. "ND" no plaques detected, limit of detection of the assay is 1 x 103 PFU/mL.
